# Rainfall-induced microbial resuscitation reveals functional decoupling across biocrust succession

**DOI:** 10.1093/ismejo/wrag231

**Published:** 2026-09-04

**Authors:** Raúl Román, Fernando T Maestre, Estelle Couradeau

**Affiliations:** Department of Ecosystem Science and Management, The Pennsylvania State University, University Park, PA 16802, United States; Instituto Multidisciplinar para el Estudio del Medio Ramón Margalef (IMEM), Universidad de Alicante, Alicante, 03690, Spain; Department of Agronomy, University of Almería, Almería, 04120, Spain; Environmental Science and Engineering, Biological and Environmental Science and Engineering Division, King Abdullah University of Science and Technology, Thuwal, 23955-6900, Kingdom of Saudi Arabia; Department of Ecosystem Science and Management, The Pennsylvania State University, University Park, PA 16802, United States; The Huck Institutes of Life Sciences, The Pennsylvania State University, PA 16802, United States

**Keywords:** biological soil crusts, BONCAT-FACS, microbial dormancy, precipitation pulse, dryland microbiomes, active microbial community, soil microbial ecology

## Abstract

Dryland ecosystems rely on infrequent rainfall pulses to activate soil microbial communities, yet the fraction and identity of microbes resuscitating after hydration remain unclear. We applied bioorthogonal non-canonical amino acid tagging coupled with fluorescence-activated cell sorting (BONCAT-FACS) and 16S rRNA gene sequencing to identify translationally active bacteria in early- (L-BSC) and late (D-BSC) successional cyanobacteria-dominated biocrusts subjected to 3 mm simulated rainfall under light and dark conditions. Our results reveal that only a small subset of the microbial community resumes activity within six hours, with higher active cell abundances in mature crusts. Microbial activity patterns were largely independent of light exposure and showed partial decoupling from total community composition, indicating that presence does not predict short-term function. These findings suggest that biocrust maturity shapes microbial activation dynamics and that functional responses to precipitation pulses are governed by a conserved pool of fast responders, informing predictions of dryland soil microbiome resilience under changing precipitation regimes.

## Introduction

In drylands, the largest terrestrial biome on Earth, water scarcity constrains biological activity and ecosystem functioning [1]. In these systems, sporadic precipitation causes brief periods of soil microbial activity, where microbes undergo a pulse regime characterized by long dormancy phases followed by rapid activation upon wetting [2, 3]. However, it remains difficult to determine both the fraction and the identity of the desert soil microbiome that rapidly resuscitates after quiescence, limiting our ability to link soil functions to microbial activity. With climate models predicting an increased frequency of extreme precipitation events in drylands [4], understanding these microbial responses is increasingly critical for predicting soil microbiome functioning and biogeochemical processes under climate change scenarios [5].

Desert-inhabiting biological soil crusts (biocrusts) are ideal model systems for exploring coordinated microbial activity after rewetting. These are complex soil surface communities resulting from the close association of photoautotrophs (e.g. cyanobacteria, lichens, or mosses), heterotrophs, and mineral soil particles [6, 7]. Biocrusts cover ~30% of the global dryland area [8], acting as a mantle of fertility by fixing atmospheric carbon and nitrogen and incorporating these into the soil surface [9, 10]. Biocrust constituents are well adapted to the pulsed nature of drylands mainly through their capacity to survive prolonged desiccation [11, 12] and quickly transition from quiescence to activity during short hydration events [13]. This rapid resuscitation has been well characterized in cyanobacteria, which possess several adaptations for revival following hydration and for anticipating forthcoming desiccation [14–16]. In contrast, the identity and fraction of heterotrophic microbiota that quickly become active during these short wetting events remain incompletely resolved, despite growing evidence from transcriptional studies indicating that early resuscitation responses are taxon-specific [17–19].

Biocrust development often begins with surface colonization by pioneer, motile filamentous cyanobacteria such as *Microcoleus vaginatus* [20], forming light-coloured biocrusts (L-BSC). This is typically followed by the establishment of light-adapted sessile heterocystous cyanobacteria such as *Nostoc, Scytonema,* or *Tolypothrix* [21], resulting in the formation of dark-pigmented crusts (D-BSC). These successional transitions are associated with increases in microbial biomass, diversity, structural complexity, and photosynthetic and nitrogen-fixation capacities [22–24]. Because cyanobacteria rapidly resume photosynthesis within minutes after wetting under daylight [14], heterotrophic microbial activation during hydration pulses is often assumed to be closely linked to this activity [18]. Consequently, daylight rainfall events are expected to promote stronger or distinct heterotrophic activation than nocturnal events, during which photosynthesis is absent, and nitrogen fixation is diminished [25]. However, it remains unclear whether light exposure during brief rewetting events directly influences microbial activity, or if such coupling is weak or absent in the short term.

Distinguishing active microorganisms from dormant or relic populations is critical for addressing these questions, especially because bulk DNA-based measurements may capture dormant cells, relic DNA, or DNA from non-viable organisms [26]. Recently, bioorthogonal non-canonical amino acid tagging (BONCAT) has emerged as a powerful approach to probe microbial activity in various environments [27, 28]. This method labels translationally active microorganisms by incorporating non-canonical amino acids into newly synthesized proteins, allowing their subsequent recovery and identification via fluorescence-activated cell sorting (FACS) and sequencing [29, 30]. BONCAT-FACS has recently been applied to recover and identify active cells from soils [31–33], providing new opportunities to interrogate microbial resuscitation following hydration in dryland ecosystems and to assess the extent to which microbial presence, as measured by bulk DNA sequencing, reflects short-term translational activity.

Here, we used BONCAT-FACS coupled with 16S rRNA gene sequencing to identify the fraction and identity of microbes that resume translational activity within hours after a simulated rainfall event. We compared early-(L-BSC) and late-successional (D-BSC) cyanobacteria-dominated biocrusts incubated under light (Sun) or darkness (Night) to evaluate whether light exposure during rewetting alters the composition of active communities and whether this response varies across successional stages. We hypothesized that (i) mature crusts host higher total and active microbial abundances, (ii) if heterotrophic activation is linked to photosynthesis, light exposure during rewetting should shift both the fraction and composition of active populations, and (iii) if heterotrophic activation is largely independent of immediate photosynthetic activity, the composition of active populations should remain similar across light conditions and only partially track successional turnover in bulk DNA communities.

## Materials and methods

### Site and biocrust sampling description

**Figure 1:**
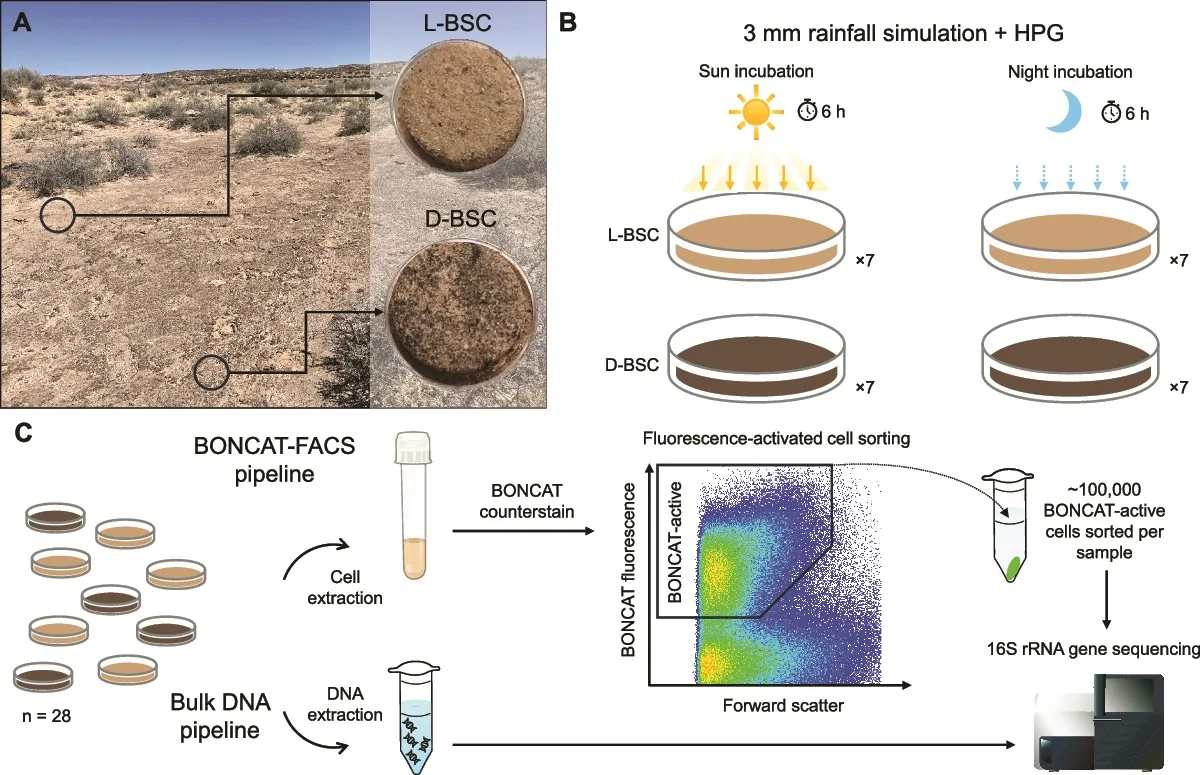
Experimental workflow. (A) Cyanobacteria-dominated biocrusts representing early (L-BSC) and late (D-BSC) successional stages were collected at the Green Butte Site (Moab, UT, USA). (B) Intact biocrust microcosms received a 3 mm simulated rainfall event containing HPG (total *n* = 28). Samples were incubated for 6 h under either illuminated (Sun incubation) or dark (Night incubation) conditions. (C) Following incubation, samples were split and harvested for parallel analyses: one underwent the BONCAT-FACS pipeline, which included cell extraction in saline buffer, HPG click reaction, and fluorescence-activated cell sorting, and the other part was used for bulk DNA extraction. Both fractions were analysed by 16S rRNA gene amplicon sequencing (see below) to determine the composition of Bulk DNA and BONCAT-active bacterial communities.

Biocrust samples were collected in April 2022 at the Green Butte Site (38.720160° N, 109.683221° W), located in Moab (Utah, USA), situated ~1350 m above sea level. The average annual precipitation is 228 mm. Biocrusts are widespread in open spaces and are primarily dominated by cyanobacteria (Fig. 1A). *M. vaginatus* often dominates in lighter, early successional crusts (L-BSC), whereas *Microcoleus steenstrupii* is found in more mature, darker crusts (D-BSC) dominated by heterocystous cyanobacteria such as *Nostoc, Scytonema*, and *Tolypothrix* [34]. Fourteen replicates of each biocrust maturity type (*n* = 28) were collected using circular sterile containers (2.5 cm diameter, 1 cm depth), air-dried, transported to Penn State University (Pennsylvania, USA), and stored in the dark at (20% RH) with drierite until experimentation.

### Biocrust wet-up experiment

Each biocrust microcosm (~4–5 g) was pre-acclimated at 25°C for one week under dry conditions in a growth chamber to a 12:12 h light/dark cycle before rainfall simulation to standardize biocrust physiological state and reduce variability related to collection, storage, and previous environmental history. During the light phase, samples were illuminated with 400 μmol photons m^−2^ s^−1^ photosynthetically active radiation. Following acclimation, each microcosm was subjected to a 3 mm rainfall event to simulate a low-intensity pulse that closely matches the upper end of the most frequent precipitation range recorded at the study site (0.25–2.87 mm). Samples received 1.36 mL of sterile 1 mM L-homopropargylglycine (HPG) solution (Click Chemistry Tools; Scottsdale, AZ, USA), which corresponds to ~77.5% of the soil water-holding capacity. Half of the microcosms were incubated under light conditions (Sun incubation), whereas the other half were incubated in complete darkness (Night incubation). Samples were incubated at 25°C for 6 h (Fig. 1B), a time interval expected to capture translational activation of existing ribosomes and to minimize the effect of new biomass generation [35]. Following incubation, microcosms were divided into two subsamples: one for counting viable cells and probing microbial activity through the BONCAT-FACS pipeline, and the other for bulk DNA extraction (Fig. 1C). At harvest, the subsample undergoing the BONCAT-FACS pipeline was transferred to a 15 mL conical tube containing 5 mL of 0.02% Tween 20 in 1× PBS and vortexed at 3000 rpm for 5 min to extract cells. The supernatant containing cell suspensions was stored at −20°C in 10% glycerol until further processing. Biocrust subsamples used for retrieving bulk DNA were flash-frozen and stored at −80°C.

### Probing microbial activity with BONCAT

BONCAT was used to track translational activity within the biocrust microbiome by incorporating HPG into newly synthesized proteins. A subsample aliquot was thawed at 4°C for 1 h and processed following a modified click-chemistry protocol adapted from [28]. Samples were gently vortexed and centrifuged at 500 × g for 5 min to pellet soil particles, after which the supernatant was recovered and passed through a 35 μm cell strainer to remove residual debris. Cells were then pelleted by centrifugation at 14000 × g for 5 min and resuspended in 300 μL of 1× PBS, followed by a brief vortex to ensure thorough mixing. Subsequently, a copper-catalyzed click reaction was conducted to bind the HPG to a fluorescent probe (FAM picolyl azide dye, Ex/Em 490/510 nm) by adding 200 μL of a reaction mix to each sample and incubating for 30 min at room temperature in the dark. The reaction mix consisted of 5 μL CuSO_4_ (100 μM final concentration), 10 μL tris-hydroxypropyltriazolylmethylamine (THPTA, 500 μM final concentration), and 3.3 μL FAM picolyl azide dye (5 μM final concentration), buffered with 50 μL freshly prepared 5 mM sodium ascorbate and 50 μL 5 mM aminoguanidine HCl in 1× PBS, and brought to 880 μL with PBS. Following incubation, excess dye was washed off by centrifugation at 14000 × g for 5 min. Pellets were washed three times with 1 mL of 1× PBS and finally resuspended in 500 μL PBS for downstream processing.

### Flow cytometry and sorting of translationally active cells

The proportion of translationally active cells (BONCAT-active) relative to the total microbial community was quantified using flow cytometry on a BD LSRFortessa Cell Analyzer (Becton, Dickinson and Company). To distinguish viable cells from soil particles, broken cells, debris, and free DNA, samples were stained with SYTO59 DNA counterstain (0.5 μM; Invitrogen; Ex/Em 622/645 nm) for 5 min in the dark. Instrument thresholds were set to detect particles larger than 0.2 μm using calibration beads (Invitrogen). The instrument was set up to capture the BONCAT fluorescence via the FAM picolyl azide dye on the green channel, using a 488 nm blue laser, and SYTO59 fluorescence in the red channel using a 630 nm red laser. Viable cells were identified by gating SYTO59-positive events against unstained control. BONCAT-active cells were then enumerated from within the SYTO59-positive population using a nested gate on the green channel. A water-incubated and HPG-free control sample was used to define background fluorescence associated with the click reaction on the green channel, and the false-positive recovery was set at 0.2%. Total cell density was subsequently estimated on the same instrument by counting SYBR Green-positive events (0.5 μM; Invitrogen; Ex/Em 498/522 nm) per microliter, using unstained samples as a control. For DNA extraction and sequencing, around 100 000 BONCAT-active cells per sample were sorted on a MoFlo Astrios Cell Sorter (Beckman Coulter). To ensure consistency during cell sorting, the same fluorophores, excitation wavelengths, controls, and predefined gating strategy established on the BD LSRFortessa were applied during FACS, following established protocols [33].

### DNA extractions and 16S rRNA gene amplicon sequencing

We extracted bulk total DNA from 250 mg of dry biocrust sample with the DNeasy PowerSoil Pro Kit (Qiagen). To enhance DNA collection, samples were added to bead tubes and disrupted at 30 Hz for 1 min in a Tissuelyser II using a tube adaptor (QIAGEN, Aarhus, Denmark). Our BONCAT-active library preparation was adapted from [28]. Sorted cells were centrifuged at 14000 × g for 10 min at 4°C. Subsequently, the supernatant was removed by gentle aspiration. The pelleted cells were lysed using PrepGEM (zyGEM, Charlottesville, VA, USA) chemical lysis in 5 μL reactions, following the manufacturer's instructions. Subsequently, tubes were vortexed for 1 min to detach the cells from the tube walls, and pulse centrifuged at 500 × g. The 5 μL mix was transferred to 0.2 mL PCR tube strips and incubated in a thermocycler at 37°C for 30 min, followed by 75°C for 40 min.

The 515f-Y (GTGYCAGCMGCCGCGGTAA, [36]) and 806R-B (GGACTACNVGGGTWTCTAAT, [37]) primers were used to amplify the 16S rRNA gene from BONCAT-active cell lysate and bulk total DNA. PCR amplification occurred in a final volume of 25 μL, including 12.5 μL of the 2× Platinum SuperFi PCR Master Mix, 1.25 μL of the forward primer (at 10 μM), 1.25 μL of the reverse primer (at 10 μM), 5 μL of 5× SuperFi GC Enhancer, and 5 μL of cell lysate or DNA template. The PCR conditions involved an initial denaturation step at 98°C for 2 min, followed by 30 cycles consisting of a 10 s denaturation step at 98°C, a 20 s annealing step at 55.4°C, a 15 s elongation step at 72°C, and a final elongation step of 5 min at 72°C. Library preparation and indexing were conducted at The Huck Life Science Genomics Core. Libraries were sequenced on a MiSeq System (Illumina) using 2 × 250 bp paired-end sequencing with 15% PhiX.

### 16S rRNA gene amplicon-sequencing data processing

Demultiplexed FASTQ files were processed with Cutadapt (v1.18, [38]) to remove primers. Trimmed reads were processed in R using DADA2 (v1.26, [39]). After quality profile inspection, forward and reverse were truncated at 230 and 200 base pairs (bp), respectively. After truncation, reads with more than two (forward) or five expected errors (reverse) were discarded (maxEE = c(2,5)), and reads with ambiguous bases were removed. After sequence inference and chimera removal (method = consensus) and to reduce redundancy at the ASV level, ASV sequences were oriented and aligned in DECIPHER (v3.4.0, [40]) and clustered at 97% identity using hierarchical clustering (TreeLine, UPGMA, cutoff = 0.03), selecting the most abundant ASV per cluster as the representative sequence. Subsequently, we used the DADA2 native naïve Bayesian classifier method to assign taxonomy, pre-trained on the SILVA database (v138.2_toGenus). ASVs belonging to chloroplasts or mitochondria were removed. A subset containing *Cyanobacteriota* was reserved for the exploration of relative abundance between biocrust types and fractions. Due to the known limitations of flow cytometry in capturing large filamentous organisms [32] we filtered out *Cyanobacteriota* for the rest of the bioinformatic analyses.

### Statistics of microbial community composition

We conducted the statistical analysis in R (v4.5.1, [41]). The filtered dataset was used to test differences in microbial composition across fractions (Bulk DNA vs BONCAT-active), biocrust type, and incubation conditions. Bray-Curtis dissimilarities between samples were calculated from relative abundance tables using the vegdist() function of the R package vegan (v2.6–4, [42]) and visualized using a non-metric multidimensional scaling (NMDS) ordination. The effects of individual factors and their interactions on community composition were tested using a PERMANOVA via the function adonis2() from the same package. β-dispersion differences were quantified using the Euclidean distance of each sample to its group centroid to test for significance among groups. Distances were extracted from the results output and fit to a linear model to evaluate each factor individually, as well as all pairwise interactions among them. Estimated marginal means (EMMs) were computed from that model using the emmeans R package (v2.0.3, [43]), and pairwise differences among EMMs were tested using Tukey HSD post-hoc comparisons. The relative abundance of bacterial phyla was depicted using a stacked bar plot using the ggplot2 package (v3.5.1, [44]).

Prior to differential abundance analyses, the dataset was filtered to remove extra rare taxa by keeping only those that: (i) were present in at least 3 samples, and (ii) had at least 50 reads. Differential abundance analyses were subsequently conducted using ANCOM-BC2 (v2.2.1, [45]) implemented in R. To evaluate the effect of incubation conditions (Sun vs Night) on ASV differential abundances, we first fitted interaction models between fraction (BONCAT-active vs Bulk DNA) and incubation conditions separately for each biocrust type, and between biocrust type and incubation conditions separately for each fraction. Models were fitted using FDR adjustment (q < 0.05), pseudo_sens = T, struc_zero = F, and the rest of the parameters set to default. Because none of these analyses showed significant effects of incubation conditions or interaction terms after FDR adjustment, the data from both incubation conditions were pooled for all subsequent analyses. Subsequently, fixed-effect models were fitted at the ASV level for (i) the BONCAT-active – Bulk DNA contrast within each biocrust type level and (ii) the D-BSC – L-BSC contrast within each fraction level. The resulting significant log-fold-changes (LFC) estimates were extracted. To evaluate the consistency of microbial responses across contrasts, LFC values were compared between the two levels of each contrast using scatterplots, including those ASVs that were significantly enriched or depleted in one or both levels of the contrast (q < 0.05, diff_robust = TRUE, and passed_ss = TRUE). Spearman correlations were calculated exclusively for the ASVs that were significant in both levels of each contrast to quantify concordance in the magnitude and direction of the enrichment or depletion.

## Results

### Differences in microbial abundance and activity across biocrust types and incubation conditions

**Figure 2:**
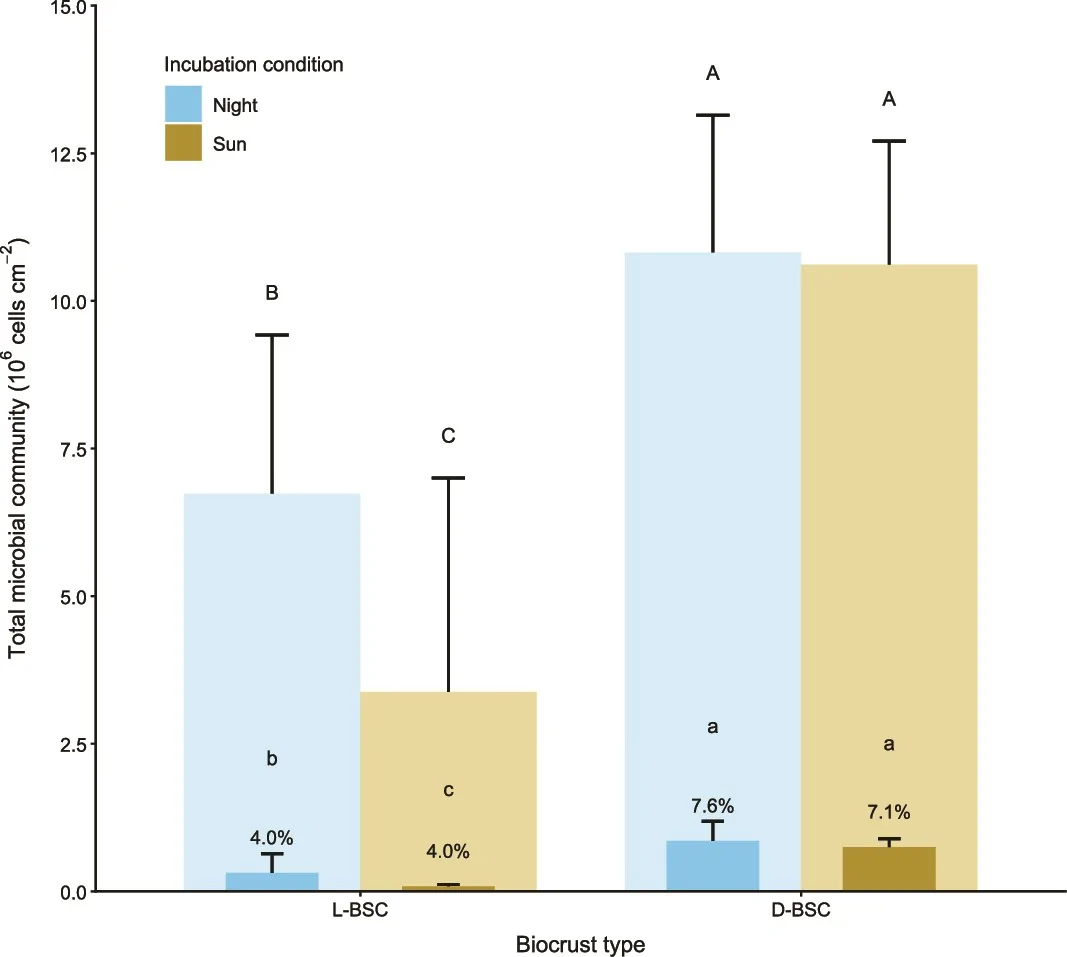
Mean abundance of total and BONCAT-active microbial cells in both biocrust types. Bars represent the total microbial community (semi-transparent) with overlaid BONCAT-active fractions (solid bars). Upper error bars represent SD. Percentages above error bars correspond to the mean proportion of BONCAT-active cells relative to the total community. Capital and lowercase letters indicate significant differences (ANOVA, *P* < .05) among incubation conditions and biocrust types for total and active fractions, respectively. All test results are in Tables S1 and S2 of the Supplementary Materials.

The abundance of total and active cells differed significantly between biocrust types and incubation conditions (Fig. 2). Total microbial abundance was consistently higher in D-BSC compared to L-BSC under both incubation conditions, reaching up to 3-fold higher values in D-BSC incubated under Sun. Within L-BSC, the Night incubation exhibited higher cell abundances than the Sun incubation, whereas no significant differences between incubation conditions were found in the D-BSC (Table S1). For BONCAT-active cells, the biocrust type had a strong effect (Table S2, *F* = 34.3, *P* < .001), whereas neither incubation conditions nor the interaction had significant effects (*P* = .125 and *P* = .545, respectively). Active cell abundances were higher in D-BSC than in L-BSC, showing a proportion of BONCAT-active cells around 7.1%–7.6% of the total community in D-BSC, compared to ~4.0% in L-BSC. Active cell abundances tended to be higher under Night than Sun incubation in L-BSC, but this pattern was absent in D-BSC.

### Fraction and biocrust type as main drivers of microbial community structure

**Figure 3:**
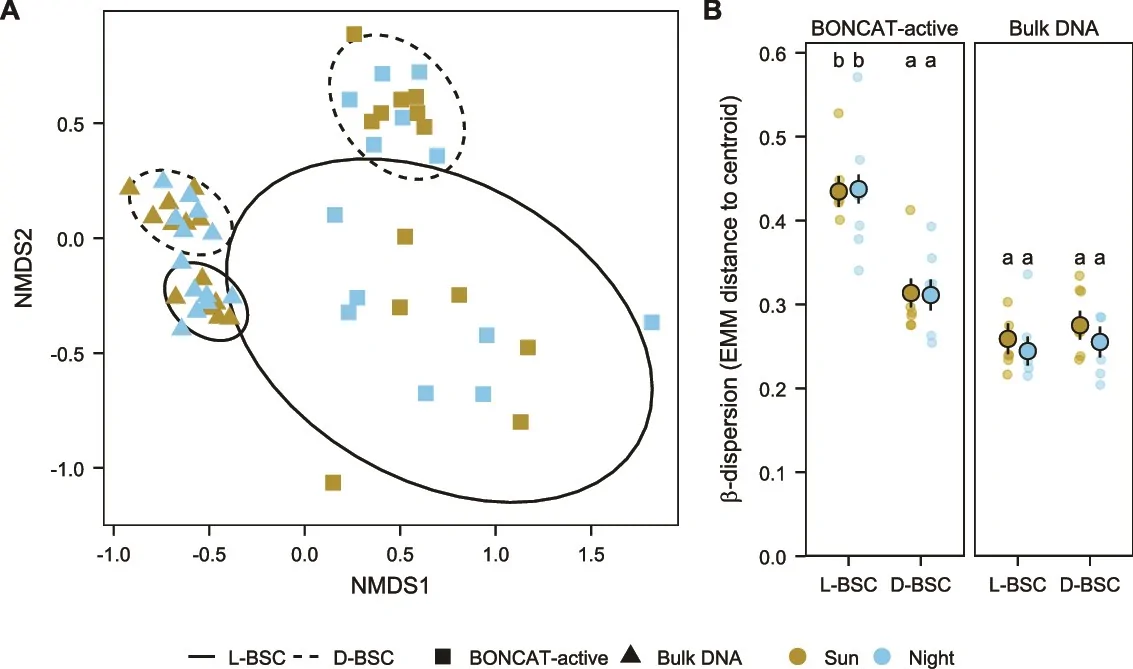
Microbial community structure and *β*-dispersion across biocrust types, fractions, and incubation conditions. **(**A) Non-metric multidimensional scaling (NMDS) ordination based on Bray–Curtis dissimilarities showing bacterial community composition by incubation condition (Sun vs Night), with point shapes indicating fraction (BONCAT-active vs Bulk DNA). The 95% confidence ellipses represent biocrust type (L-BSC: solid; D-BSC: dashed). Cyanobacteria were excluded from the ordination due to low representation in the BONCAT-active fraction (Fig. 4) and to reduce bias associated with large filamentous organisms. (B) Estimated marginal means (EMMs) of *β*-dispersion, calculated as the Bray–Curtis dissimilarity of each sample to its group centroid, error bars represent ±SE, and letters indicate pairwise group differences based on the Tukey HSD post-hoc test.

We used BONCAT-FACS combined with 16S rRNA gene sequencing to identify the active members of the microbial community in L-BSC and D-BSC. NMDS based on Bray–Curtis dissimilarity showed a clear separation between BONCAT-active and Bulk DNA fractions, particularly in D-BSC (Fig. 3A). Biocrust type also influenced community composition, but the separation between biocrust types was more pronounced in the Bulk DNA fraction. Samples incubated under Sun and Night grouped similarly, indicating that incubation conditions had no significant effect on community composition. PERMANOVA results supported these observations, showing that fraction and biocrust type significantly shaped bacterial composition, explaining 30.0% and 13.0% of the variation, respectively (Table 1). In contrast, incubation condition was not significant, accounting for only about 1% of the variation. Additionally, a significant interaction between fraction and biocrust type indicated that the effect of biocrust type on community composition differed between BONCAT-active and total community DNA fractions, consistent with the NMDS ordination patterns. Incubation treatment did not significantly affect the within-group variation (*β*-dispersion) of biocrust microbiomes (ANOVA, *F* = 0.5, *P* = .40). *β*-Dispersion was equal between L-BSC and D-BSC in the bulk DNA fraction, whereas it differed significantly in the BONCAT-active fraction, with L-BSC showing significantly higher *β*-dispersion than D-BSC (Fig. 3B).

**Table 1:** PERMANOVA results based on Bray–Curtis dissimilarities testing the effects of microbial fraction, biocrust type, incubation conditions, and their pairwise interactions on microbial community composition.

Factor	*P*	*R* ^2^	*F*
Fraction	.001^*^	0.30	28.9
Biocrust type	.001^*^	0.13	12.4
Incubation conditions	.579	0.01	0.8
Fraction × Biocrust type	.001^*^	0.07	6.7
Fraction × Incubation conditions	.663	0.01	0.8
Biocrust type × Incubation conditions	.632	0.01	0.8

Asterisks (^*^) indicate *P*-values where factors significantly impacted microbial community composition, while *R*^2^ values reflect the percentage of variation explained by each experimental factor.

### Differentially abundant taxa between BONCAT-active and Bulk DNA fractions

**Figure 4:**
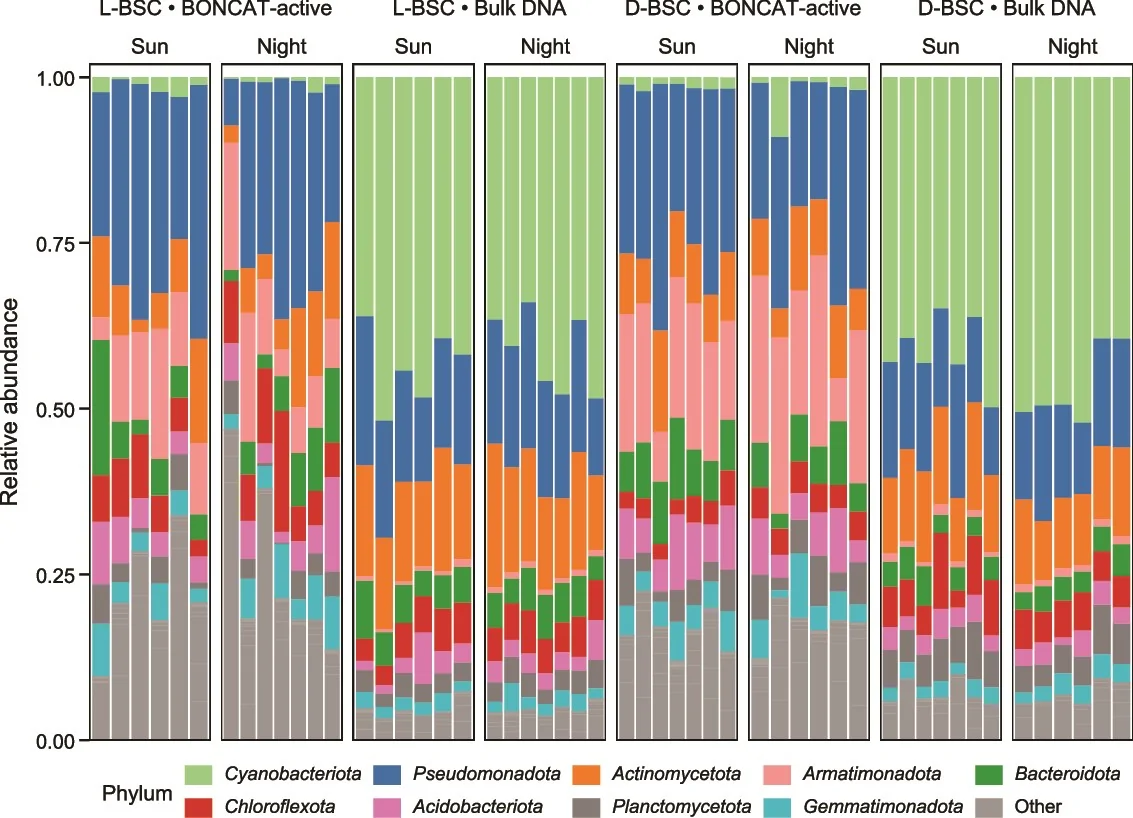
Relative abundance of the ten most abundant bacterial phyla across all samples. Phyla not belonging to the ten most abundant were grouped as Other. Each stacked bar is a sample. Relative abundances were calculated from unrarefied ASVs.

Because light exposure had no significant influence on microbial structure, both incubations were pooled together to analyse the microbial composition between fractions. The relative abundance of the most abundant phyla differed significantly between fractions in both biocrust types. In the Bulk DNA fraction, *Cyanobacteriota, Pseudomonadota, Actinomycetota*, and *Chloroflexota* were the most abundant in both biocrust types, accounting for over 60% of all reads (Figs 4, S1, and  S2), suggesting that many microbial cells assigned to these phyla were either dormant or not viable. Conversely, *Armatimonadota* was more abundant in the BONCAT-active fraction in both biocrusts, whereas *Abditibacteriota* was significantly higher only in L-BSC (Figs S1 and S2).

**Figure 5:**
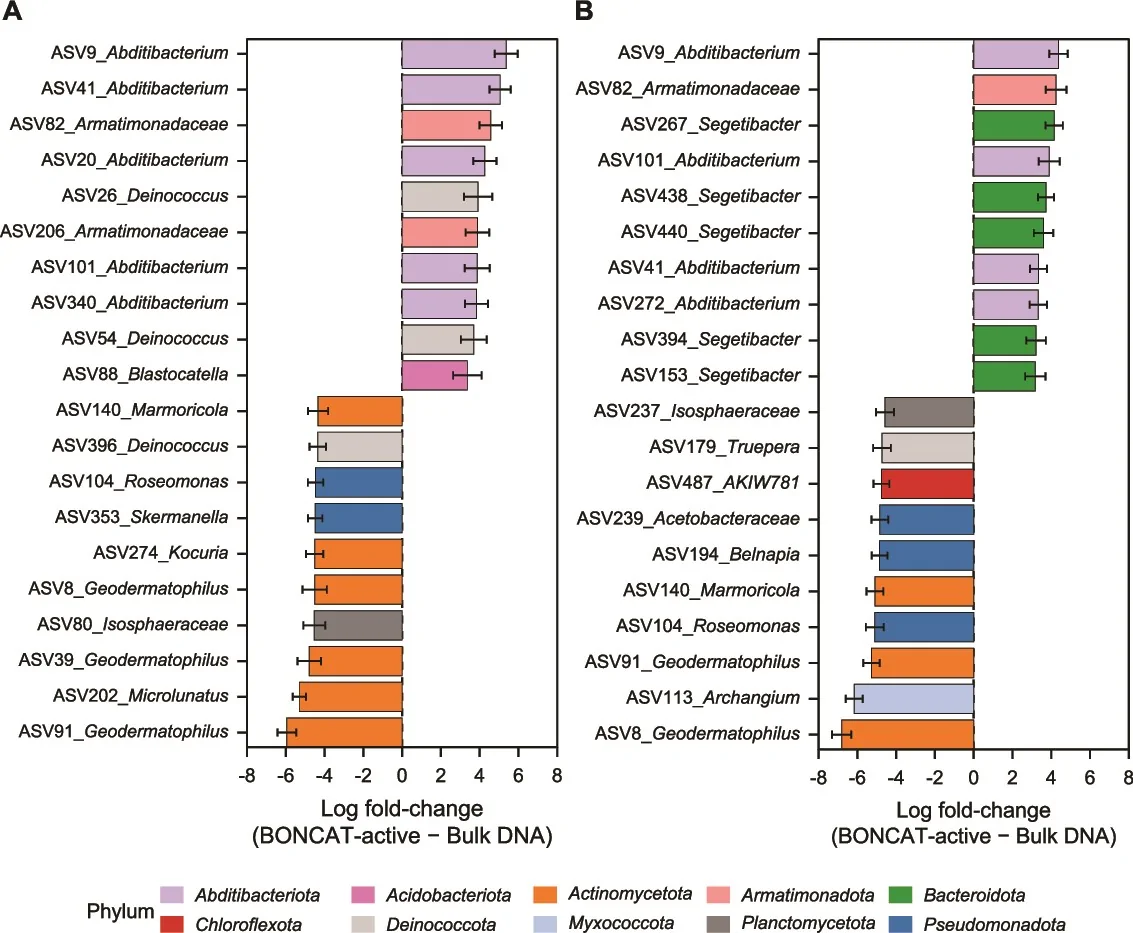
Top 10 differentially enriched ASVs in BONCAT-active versus bulk DNA fractions across biocrust types: (A) Light cyanobacteria-dominated (L-BSC) and (B) Dark cyanobacteria-dominated (D-BSC). ASVs were selected by the largest absolute LFC for the BONCAT-active versus bulk DNA contrast. Positive LFC values indicate enrichment in the BONCAT-active, whereas negative values indicate enrichment in the Bulk DNA fraction. Error bars represent ±SE. Only ASVs with significant differential abundance after FDR Benjamini–Hochberg correction (q < 0.05) are shown.

To determine which taxa drove these compositional differences, we applied ANCOM-BC2 to identify differentially abundant ASVs between fractions (q < 0.05). Across both biocrusts, 488 ASVs significantly differed between fractions, including 102 enriched in the BONCAT-active and 386 enriched in the Bulk DNA fraction (full list in Table S3). ASVs enriched in the Bulk DNA fraction were dominated by *Actinomycetota*, which accounted for ~68.1% (263 of 386) of all ASVs in the Bulk DNA fraction. Within this phylum, enrichment was concentrated in a few genera, including *Rubrobacter, Nocardioides, Pseudonocardia, Propionibacterium, Geodermatophilus*, and *Blastococcus* (Fig. 5, negative LFC). Additional ASVs enriched in this fraction belonged to *Pseudomonadota* (15.3%; 59 ASVs), *Chloroflexota* (6.0%; 23 ASVs), and *Bacteroidota* (5.2%; 20 ASVs). Conversely, most BONCAT-active ASVs belonged to *Abditibacteriota, Bacteroidota*, and *Deinococcota* in both biocrust types. Within these, activity was concentrated in a small number of genera, including *Abditibacterium, Segetibacter*, and *Deinococcus*, which contained multiple ASVs strongly enriched in the BONCAT-active fraction of both biocrust types, and minor enrichments by *Sphingomonas* and *Solirubrobacter* (Fig. 5, Table S3).

### Concordance between translational activity and community composition

**Figure 6:**
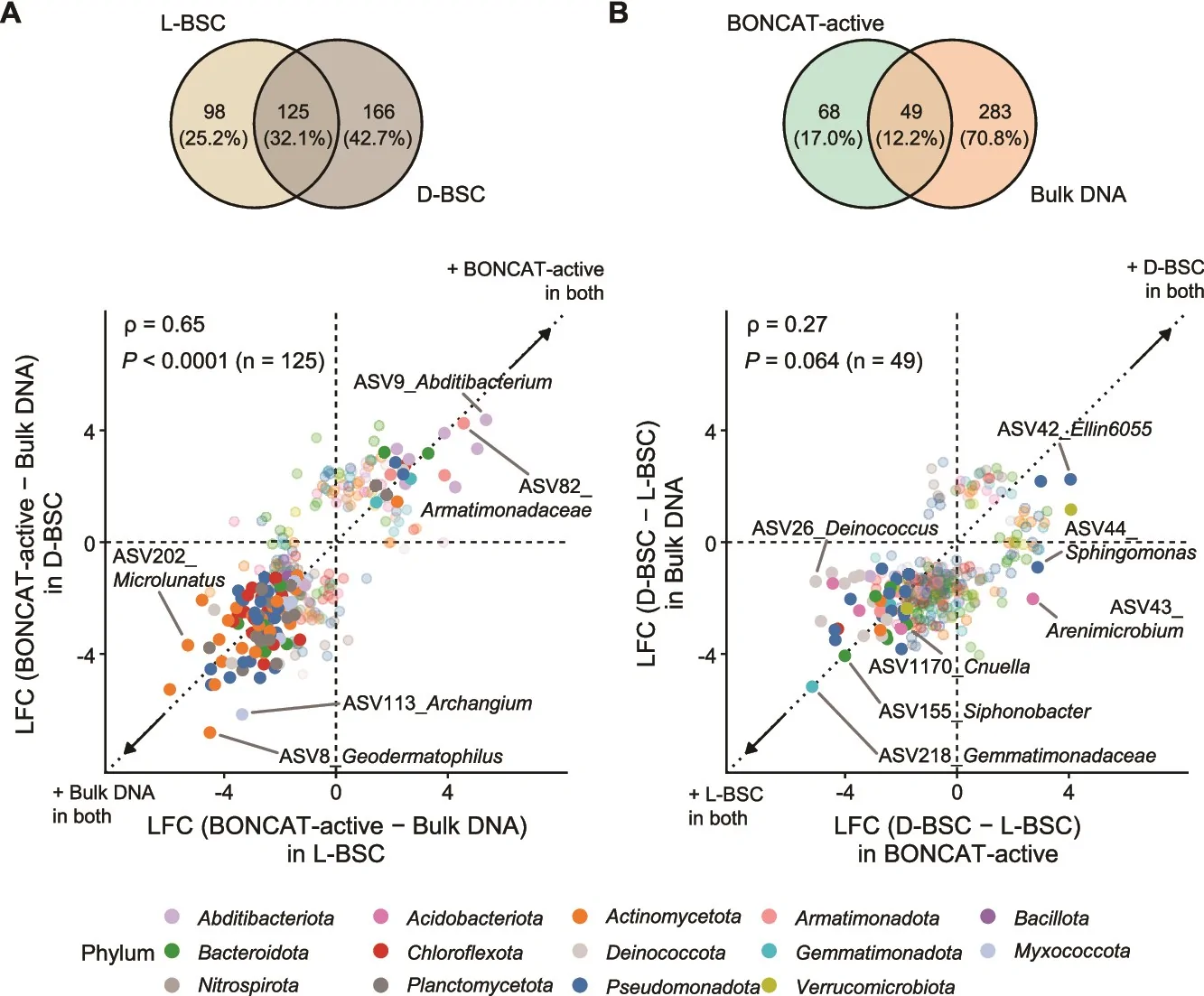
Concordance of differential enrichment patterns across biocrust maturity and activity fractions. Scatterplots compare ANCOM-BC2 log fold-change (LFC) estimates for individual ASVs (each point) between pairs of contrasts. (A) Comparison of the BONCAT-active vs bulk DNA contrast across successional stages: the x-axis shows LFCs in L-BSC, and the y-axis shows LFCs in D-BSC for the same ASVs. Positive LFC values indicate ASVs enriched in the BONCAT-active fraction, whereas negative values indicate enrichment in the bulk DNA fraction. (B) Comparison of the D-BSC vs L-BSC contrast between fractions: the x-axis shows LFCs estimated from the BONCAT-active fraction, and the y-axis shows LFCs from bulk DNA. Positive LFC values indicate ASVs enriched in D-BSC (mature) relative to L-BSC (early), whereas negative values indicate enrichment in L-BSC. In both panels, ASVs significant in at least one of the two compared conditions (q < 0.05) are shown; light-coloured points are significant in only one condition, and dark-coloured points are significant in both. The horizontal and vertical zero lines separate enrichment directions, and quadrants summarize concordant (top-right, bottom-left) versus divergent (top-left, bottom-right) responses. Venn diagrams show the overlap in significant ASVs between the compared conditions. Spearman correlations (ρ) quantify agreement in enrichment magnitude and direction using only ASVs significant in both conditions.

To evaluate whether the taxa enriched in different fractions were consistent across biocrust developmental stages, we compared the LFC estimates for the BONCAT—Bulk DNA contrasts between L-BSC and D-BSC crusts (Fig. 6A, Table S3). Approximately one third of the ASVs showed concordant direction and magnitude of change in both biocrusts, clustering along the 1:1 diagonal and indicating that the same taxa tended to be either translationally active (positive LFC) or present in the bulk community (negative LFC) regardless of biocrust maturity (Spearman ρ = 0.65, *P* < .001). However, the specific composition of active taxa shifted along the successional gradient, with only 21.4% (18 of 84) of ASVs enriched in the BONCAT-active shared between L-BSC and D-BSC. In contrast, a larger proportion of ASVs enriched in the Bulk DNA fraction was shared between L-BSC and D-BSC (36.4%).

To test whether compositional changes measured in the Bulk DNA community along the successional gradient produced parallel responses in translational activity, we compared D-BSC – L-BSC contrast between BONCAT-active and Bulk DNA fractions (Fig. 6B, Table S4). Only a small group of ASVs (12.2%) showed concordant responses between fractions, and the relationship between them was weak and not significant (Spearman ρ = 0.27, *P* = .064), revealing that total community shifts linked to biocrust maturity were not reflected in equivalent changes in active taxa. Nevertheless, a few distinct outliers showed contrasting response types. For example, members of the genus *Ellin6055* showed positive LFCs in both fractions, suggesting an increase in both activity and abundance with maturity, whereas *Cnuella, Massilia, Noviherbaspirillum*, and members of *Gemmatimonadaceae* increased presence and activity in early-successional biocrusts (negative LFCs). However, the scattered distribution of points and low fraction concordance confirm that most taxa vary independently in abundance and activity, supporting the idea that bulk microbial presence and short-term metabolic activity signatures are partially decoupled across the biocrust successional gradient.

## Discussion

### Microbial abundance and activity are controlled by biocrust maturity

Consistent with our first hypothesis, both total and active cell abundances differed between biocrust developmental stages. D-BSC exhibited higher cell abundances than L-BSC, consistent with previous findings that report increased cell abundance with greater photoautotrophic biomass [46]. Our study builds on previous research and demonstrates that biocrust maturity not only increases microbial abundance but also hosts larger populations of translationally active members. Increased microbial activity may also help explain the higher respiration rates consistently reported in more developed biocrusts [46–48]. These functional differences are typically attributed to greater organic carbon and microbial biomass. However, our results suggest that differences in the size of the active fraction may also contribute to these functional differences, given that BONCAT-active cell counts are positively correlated with respiration rates in soils [35].

Despite these differences, only a small fraction of the community (4%–7%) resumed translational activity within the 6-hour pulse (Fig. 2). Nevertheless, these results are well above the proportions reported in bulk soil and rhizosphere using the same methodology [33]. Recent studies combining single-cell ^2^H_2_O labelling and NanoSIMS imaging suggest a much faster and more extensive resuscitation of the heterotrophic community in biocrusts, with over 75% of cells showing anabolic activity after the same time interval [49]. Although these findings indicate that most cells resume minimal repair or maintenance activity after hydration, our results show that only a small subset engages in detectable translational activity during brief wetting. Because BONCAT-FACS detects translational activity of cells not necessarily undergoing division, our approach is well-suited to capture early activation dynamics that otherwise might not be detected by growth-based methods. Looking forward, this methodology can be applied to identify rare and active species that may disproportionately contribute to short-term metabolic fluxes in biocrusts [50], an outstanding research question in the field that has remained unanswered.

### Short-term microbial activation is decoupled from real-time photosynthetic activity

Contrary to our second hypothesis, light exposure had negligible effects on microbial composition and activity, indicating that the fraction and identity of the taxa that reactivate during short-term wetting are decoupled from cyanobacterial photosynthetic activity. This asynchronous response is somewhat unexpected and challenges the prevailing view of interdependent resuscitation between photoautotrophs and heterotrophs in cyanobacteria-dominated biocrusts [51, 52]. However, we cannot rule out the possibility of light-dependent coupling in the uppermost biocrust layer, the photic zone (0–1 mm depth), which is an O_2_ supersaturated environment [12]. In this zone, bundle-forming cyanobacteria actively attract copiotrophs and diazotrophs, facilitating carbon-for-nitrogen mutualistic exchanges that could enable coupled activity over larger timescales [15, 53]. Further research is needed to elucidate these microscale metabolic interactions, for example, by using BONCAT to identify translationally active members of the cyanosphere and track proteomic changes across diel cycles [54].

### BONCAT-FACS reveals early responding taxa across biocrust succession

The clear separation between fractions observed in the NMDS (Fig. 3A) reveals a strong distinction between presence and activity in both biocrust types. The large compositional differences observed between L-BSC and D-BSC in the Bulk DNA fraction were expected and align with prior work showing clear taxonomic changes along cyanobacteria-dominated maturity gradients [34, 55]. The significant interaction between fraction and biocrust type (Table 1) reveals that biocrust maturity modulates the relationship between bulk presence and activity. As crusts mature, their active communities diverge more from their total DNA pool (Fig. 3A). This separation may be attributable to increasing cyanobacterial diversity and associated heterotrophic community complexity [46], stronger vertical stratification, and increased accumulation of dormant cells or relic DNA over time in mature crusts, a common phenomenon in soils [26]. Additionally, *β*-dispersion of BONCAT-active populations was significantly lower in D-BSC than in L-BSC, suggesting that mature communities tend to converge in their composition of active bacteria, whereas L-BSC exhibits a more heterogeneous composition [56].

Our analyses found that Bulk-enriched ASVs mostly belonged to *Actinomycetota*, which have been previously found in many other biocrust communities [17, 19, 57]. Whereas a few ASVs belonging to *Actinomycetota* were also enriched in the BONCAT-active fraction, their much higher abundance in the Bulk fraction of both crusts suggests that many members of this phylum (e.g. *Geodermatophilus, Nocardioides, Pseudonocardia*) exhibit a slow response to wetting or remain dormant during short rainfall pulses, consistent with previous reports [32]. This group includes well-known oligotrophic bacterial genera ubiquitous to cyanobacteria-dominated biocrusts [58, 59] but are typically absent from the cyanosphere [15, 60], which may partially explain their reduced activity during short-term hydration. *Actinomycetota* also exhibits physiological adaptations to desiccation, such as sporulation [61], which allows for extremely long dormant periods at the cost of a very long pulse to enable germination [62]. In contrast, the BONCAT-active fraction was predominantly enriched by *Abditibacteriota, Bacteroidota*, and *Deinococcota* in both biocrusts. *Abditibacterium*, the first cultivated representative of the candidate phylum FBP isolated from Antarctic mineral soils [63], showed a strong enrichment in the BONCAT-active fraction of both L-BSC and D-BSC, identifying this genus as an active member of the biocrust community. Other active genera, including *Cnuella, Massilia*, or *Noviherbaspirillum*, were preferentially enriched in L-BSC (Table S3). The same taxa have been found to respond to short rainfall pulses (5 h) in very distant geographical early-successional biocrusts from the Chihuahuan Desert [64], suggesting that they have specific adaptations to preserve cell structure after recurrent hydration-desiccation and preparedness for resuscitation [49].

Our observations are consistent with a general phylogenetic organization of nimble vs torpid responders in biocrusts postulated in the expanded pulse-reserved paradigm of Garcia-Pichel and Sala [65]. They also align with recent evidence showing that members of *Actinomycetota* were mainly associated with long-pulse responder strategies, whereas *Cnuella, Massilia*, or *Noviherbaspirillum* were associated with the short-pulse strategies [64]. Our BONCAT-FACS builds on this framework by directly identifying taxa that rapidly resume protein synthesis following short hydration pulses. Now that BONCAT-live permits the isolation and cultivation of BONCAT-active sorted cells [66], future research could focus on culturing early and late responders to further confirm these differences in life strategy.

### Activity patterns are consistent across biocrust maturity but decoupled from total community structure

Consistent with our hypothesis, we found a weak correlation between presence and activity across biocrust development (Fig. 6B, Spearman ρ = 0.27, *P* = .064), indicating partial functional decoupling between bulk microbial abundance and translational activity. This decoupling demonstrates that dominant or persistent taxa in the bulk community are not necessarily those responding to short rehydration, making presence alone a poor predictor of short-term functional responses in biocrusts. In contrast, a substantial portion (>20%) of the active population was shared by both early and mature biocrusts, with most active ASVs clustering along the 1:1 diagonal in both crusts (Fig. 6A). These results suggest that biocrusts share a pool of fast responders that reactivate protein synthesis upon wetting, regardless of their bulk taxonomic composition, likely reflecting a form of conserved functional redundancy typical of dryland microbiomes [67]. Despite this overlap, D-BSC crusts still exhibited a larger and more diverse population enriched in their active fraction (Table S3), mainly within *Actinomycetota, Bacteroidota*, and *Pseudomonadota*, consistent with higher niche specialization and increased functional specificity and complexity [68]. Because the most frequent precipitation events in the study area fall in the 0.25–2.87 mm range, producing hydration periods comparable to our experimental conditions, these results likely capture typical resuscitation dynamics in cyanobacteria-dominated biocrusts following rainfall. Under projected shifts towards more frequent, smaller summer rainfall events [69] and increasing human pressure driving regressions from late- to early-successional biocrusts [23], these fast-responding taxa may be favoured, potentially altering nutrient cycling and other biocrust-mediated functions in dryland soils.

Our findings likely captured one component of the microbial resuscitation dynamics in dryland biocrusts. We cannot dismiss the possibility that the concatenation of large rainfall events, such as those associated with the monsoon, and concurrent changes in other environmental factors (e.g. soil temperature) may trigger the activation and proliferation of late responders, such as *Firmicutes* or *Actinomycetota* [18, 64]. Likewise, non-rainfall water inputs, such as dew, also represent an important source of moisture for biocrusts [70] and may trigger the activation of a distinct subset of the microbial community. Future studies combining BONCAT-FACS with manipulations of antecedent soil moisture, hydration pulse duration, and different types of wetting events (rainfall vs dew) could help determine whether these factors influence the recruitment of translationally active microbes, potentially providing a more comprehensive understanding of microbial resuscitation in drylands.

## Supplementary Material

Supplementary_material_wrag231

## Data Availability

Analysis scripts, visualization scripts, and primary data generated in this study are available at https://github.com/jrf979/Biocrust_resuscitation. The sequencing data are available at the National Center for Biotechnology Information (NCBI) Sequence Read Archive (SRA), under BioProject PRJNA1458593.
