# Supplementary material for: Rainfall-induced microbial resuscitation reveals functional decoupling across biocrust succession
Source: ISME J. 2026 Sep 4;20(1):wrag231. doi: 10.1093/ismejo/wrag231 (PMC13592164; doi:10.1093/ismejo/wrag231)
Supplement: Supplementary file 1 [file supplementary_material_wrag231.zip › Supplementary_Material_Microbial_resuscitation_biocrusts_wrag231.docx]

***Supplementary Material for***

**Rainfall-induced microbial resuscitation reveals functional decoupling across biocrust succession**

Raúl Román ^1,2,3^*, Fernando T. Maestre ^4^, and Estelle Couradeau ^1,5^

^1^ Department of Ecosystem Science and Management, The Pennsylvania State University, University Park, PA, USA

^2^ Instituto Multidisciplinar para el Estudio del Medio Ramon Margalef, Universidad de Alicante, Alicante, Spain

^3^ Department of Agronomy, University of Almería, 04120, Almería, Spain

^4^ Environmental Science and Engineering, Biological and Environmental Science and Engineering Division, King Abdullah University of Science and Technology, Thuwal, 23955-6900, Kingdom of Saudi Arabia

^5^ The Huck Institutes of Life Sciences, The Pennsylvania State University, PA, USA

* Corresponding author.

Raúl Román. Address: Department of Agronomy, CITE-IIB, University of Almería, Carretera de Sacramento s/n, 04120 Almería, Almería, Spain. E-mail: [jrf979@gmail.com](mailto:jrf979@gmail.com)

**Supplementary material**

**Figure S1**. Top 6 dominant phyla in L-BSC and their enrichment in BONCAT-active vs. Bulk DNA, defined as those with the highest mean relative abundance across all samples and fractions excluding *Cyanobacteriota*. The p-values are from Wilcoxon rank-sum tests. Relative abundances were calculated from unrarefied ASVs.

**Figure S2**. Top 6 dominant phyla in D-BSC and their enrichment in BONCAT-active vs. Bulk DNA, defined as those with the highest mean relative abundance across all samples and fractions excluding *Cyanobacteriota*. The p-values are from Wilcoxon rank-sum tests. Relative abundances were calculated from unrarefied ASVs.

**Table S1**. ANOVA results testing the effects of biocrust type and incubation conditions, and their interaction on total microbial cells. Asterisks (*) indicate significant effects (*P* < 0.05).

| Model: Cells_total ~ (BiocrustType + Incubation)^2 |  |  |
| --- | --- | --- |
| Factor | ***P*** | **F** |
| Biocrust type | <0.001* | 44.2 |
| Incubation conditions | 0.020* | 6.4 |
| Biocrust type × Incubation conditions | 0.021* | 6.2 |

**Table S2**. ANOVA results testing the effects of biocrust type and incubation conditions, and their interaction on BONCAT-active microbial cells. Asterisks (*) indicate significant effects (*P* < 0.05).

| Model: Cells_active ~ (BiocrustType + Incubation)^2 |  |  |
| --- | --- | --- |
| Factor | ***P*** | ***F*** |
| Biocrust type | <0.001* | 34.3 |
| Incubation conditions | 0.125 | 2.6 |
| Biocrust type × Incubation conditions | 0.545 | 0.4 |

**Table S3**. Differentially abundant ASVs between BONCAT-active and Bulk DNA fractions across biocrust types. Provided as a separate Excel file (.csv).

**Table S4**. Differentially abundant ASVs between D-BSC and L-BSC across BONCAT-active and Bulk DNA fractions. Provided as a separate Excel file (.csv).
